# Nearly diffraction-limited X-ray focusing with variable-numerical-aperture focusing optical system based on four deformable mirrors

**DOI:** 10.1038/srep24801

**Published:** 2016-04-21

**Authors:** Satoshi Matsuyama, Hiroki Nakamori, Takumi Goto, Takashi Kimura, Krishna P. Khakurel, Yoshiki Kohmura, Yasuhisa Sano, Makina Yabashi, Tetsuya Ishikawa, Yoshinori Nishino, Kazuto Yamauchi

**Affiliations:** 1 Department of Precision Science and Technology, Graduate School of Engineering, Osaka University, 2-1 Yamada-oka, Suita, Osaka 565-0871, Japan; 2 JTEC Corporation, 2-4-35, Saito-Yamabuki, Ibaraki, Osaka 567-0086, Japan; 3 Research Institute for Electronic Science, Hokkaido University, Kita 21 Nishi 10, Kita-ku, Sapporo 001-0021, Japan; 4 SPring-8/RIKEN, 1-1-1 Kouto, Sayo, Hyogo 679-5148, Japan; 5 Center for Ultra-Precision Science and Technology, Graduate School of Engineering, Osaka University, 2-1 Yamada-oka, Suita, Osaka 565-0871, Japan

## Abstract

Unlike the electrostatic and electromagnetic lenses used in electron microscopy, most X-ray focusing optical systems have fixed optical parameters with constant numerical apertures (NAs). This lack of adaptability has significantly limited application targets. In the research described herein, we developed a variable-NA X-ray focusing system based on four deformable mirrors, two sets of Kirkpatrick–Baez-type focusing mirrors, in order to control the focusing size while keeping the position of the focus unchanged. We applied a mirror deformation procedure using optical/X-ray metrology for offline/online adjustments. We performed a focusing test at a SPring-8 beamline and confirmed that the beam size varied from 108 nm to 560 nm (165 nm to 1434 nm) in the horizontal (vertical) direction by controlling the NA while maintaining diffraction-limited conditions.

Developments of X-ray sources and optical devices have enabled a number of achievements in X-ray science. X-ray sources have steadily advanced from X-ray tubes to undulators in synchrotron radiation facilities. Furthermore, the development of X-ray free-electron lasers (XFELs)^1^,^2^ and storage ring light sources with ultimate performance^3^ have had significant impacts in various scientific fields. The quality of the focusing devices used in X-ray optical systems has been drastically improved over the past few decades. By using state-of-the-art Fresnel zone plates^4^, multilayer mirrors in the Kirkpatrick–Baez (KB) geometry^5^, and multilayer Laue lenses^6^,^7^, it is possible to achieve small focuses of 10 nm or less. Such focusing devices have enabled high-resolution and high-sensitivity X-ray analyses for cutting-edge research in materials science, the biological and medical sciences, and chemistry.

Most X-ray focusing devices have fixed numerical apertures (NAs), unlike the electrostatic and electromagnetic lenses used in electron microscopes. Several benefits could be expected if NAs could be changed during experiments. One of the most distinct benefits is the ability to perform multi-functional microscopy, which combines various X-ray analysis and microscopy techniques. For example, the combination of scanning X-ray microscopy (SXM)^8^,^9^,^10^ and coherent X-ray diffractive imaging (CDI)^11^,^12^,^13^ provides the opportunities to observe fine structures by using CDI and to visualize elemental and chemical distributions by using SXM. However, most X-ray focusing devices cannot apply both of these methods together due to their different beam size requirements: SXM requires a beam as small as possible, while CDI needs a moderately sized focused beam that can illuminate an area larger than the sample size. Variable-NA focusing optics that can produce different beam sizes and divergences will thus expand the capabilities of X-ray analyses and microscopy.

Practically, the NA can be controlled by utilizing a variable aperture, such as a slit or a diaphragm, placed near the focusing optics, although this configuration results in significant photon flux loss. However, if the variable aperture is substituted with two deformable mirrors^14^,^15^,^16^,^17^,^18^ arranged in the KB configuration, it is possible to transmit the full incident flux to the final focus. NA control can be achieved simply by changing the focal lengths of the mirrors by deforming their shapes. However, to realize a substantial NA change, a large space is required. Furthermore, it is technically challenging to fabricate high-quality deformable mirrors to achieve diffraction-limited focusing: the error between the figure and the ideal shape should be smaller than 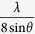, where λ and θ are the wavelength and grazing incidence angle, respectively. When λ = 0.124 nm and θ = 4 mrad, the maximum error to be accepted is ~4 nm. This severe requirement has caused substantial difficulty in achieving X-ray nanofocusing with deformable mirrors.

To overcome these problems, we developed a high-quality deformable mirror based on piezoelectric elements^18^,^19^,^20^. The test results showed that the mirror could be successfully deformed into an arbitrary elliptical shape with a shape error of less than 2.5 nm^18^. Based on the results, we proposed a variable-NA focusing system based on four deformable mirrors^21^,^22^,^23^, which is quite different from optical systems that can shape X-rays at the level of a few tens of microns or a few microns^24^. In this paper, we discuss the performance of the variable-NA focusing optical system. First, we describe a method of tuning the four deformable mirrors into a target elliptical shape using an optical interferometer and X-ray metrology. We then report on X-ray focusing experiments performed at a SPring-8 beamline with a photon energy of 10 keV. The focused beams, characterized by the knife-edge scanning method, were successfully changed by adjusting only the NA while satisfying the diffraction-limited condition.

## Results

### Variable-NA focusing system

**Figure 1:**
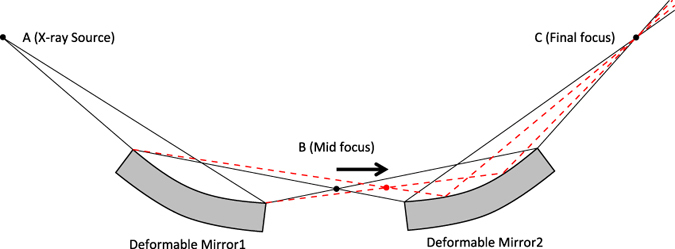
Scheme of variable-NA focusing optical system, in which NA can be controlled without changing focal plane position.

**Figure 2:**
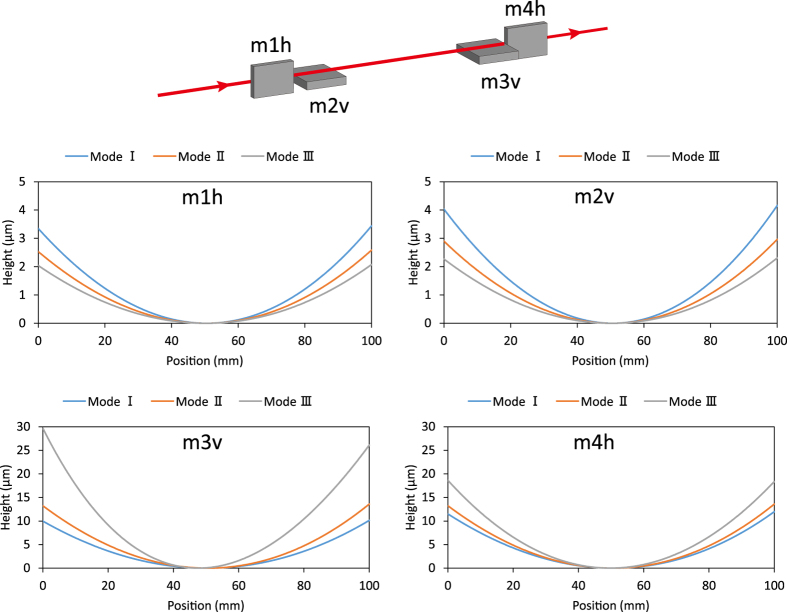
Shapes of deformable mirrors (Modes I–III).

Figure 1 depicts a one-dimensional schematic of the principle of our proposed variable-NA focusing system. In this case, the two deformable mirrors are assumed to be elliptical: the upstream mirror is designed to have foci at ‘A’ (light source) and ‘B’ (mid focus), and the downstream ellipse is designed to have foci at ‘B’ and ‘C’ (sample position). By controlling the shapes of the two mirrors, the mid focus can be shifted along the optical axis while leaving the beam axis and final focus position unchanged. These changes enable the NA of the focusing system to be varied. The design parameters of the optical system are shown in Table 1. Notably, control of the downstream mirror is more difficult when the NA is smaller, because the curvature deviation becomes significant in this case (see Fig. 2). The NA of the downstream mirror was limited to between 0.533 × 10^−3^ and 0.041 × 10^−3^ to avoid excessive deformation of the downstream mirror. We designed three focusing modes (Modes I–III), as shown in Table 2. In this experiment, the aperture ratio was defined as the ratio of the area illuminated on the downstream mirror to the maximum illumination area. Mode I had the maximum NA with an aperture ratio of 1.0, in which case the surfaces of the four deformable mirrors were fully illuminated. Mode II had an NA approximately half that of Mode I and aperture ratios of 0.5 and 0.43 in the horizontal and vertical directions, respectively. Mode III had the smallest NA with aperture ratios of 0.20 and 0.11 in the horizontal and vertical directions, respectively. The maximum (minimum) mirror deformations from a flat shape were 4 μm (2 μm) for the upstream mirrors and 30 μm (10 μm) for the downstream mirrors (see Fig. 2).

### Ultra-precise deformable mirrors

**Figure 3:**
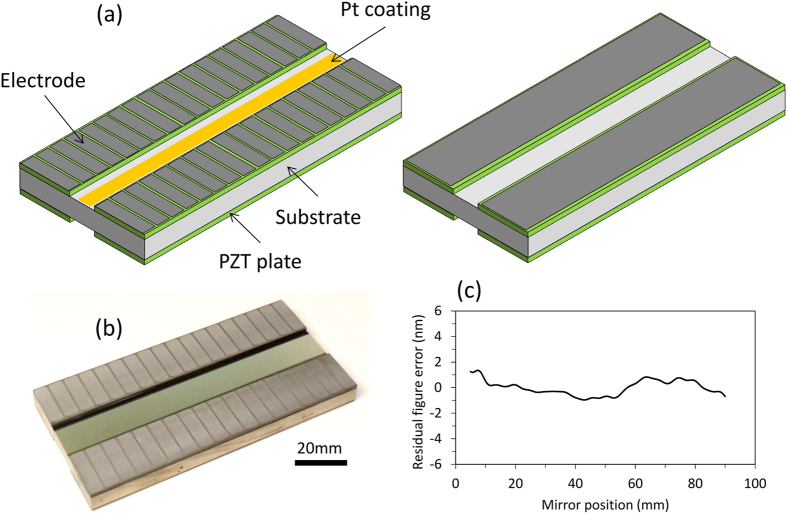
Piezoelectric deformable mirror with bimorph structure. (**a**) Schematic of deformable mirror. (**b**) Photograph of deformable mirror. (**c**) Figure errors of deformable mirrors, except for second-order polynomials.

In this study, piezoelectric deformable mirrors with bimorph structures were used (see Fig. 3). Each mirror consisted of a quartz glass substrate and four long piezoelectric plates (lead zirconate titanate, PZT), which were attached to the front and back of the substrate with glue. Bimorph structures, which are symmetric about their central layers, exhibit minimal deformations in response to temperature changes. The piezoelectric plates on the back of each substrate, which each had a single electrode produced using DC magnetron sputtering, formed the quadratic shape of the target ellipse. On the other hand, the piezoelectric plates on the front face of each substrate, which each had 18 chromium electrodes, were used to produce arbitrary shapes with a minimum spatial wavelength of 11.2 mm, which is sufficient to produce a nearly perfect ellipse. The continuous long piezoelectric plates used on the front and back enabled the deformable mirrors to reduce the surface undulations produced by the gaps between the electrodes^19^. Furthermore, the undulation could be made negligible by placing the effective reflection area at the centre of the substrate, away from the piezoelectric plates^19^. High-spatial-frequency waviness on the substrates that could not be corrected by deformation was directly removed by computer-controlled elastic emission machining (EEM)^25^ (see Methods section). In addition, middle-spatial-frequency shapes, except for the second-order-polynomial shapes, were removed by the EEM processing to lower the voltages required to form the ideal shapes. The errors of the shapes, except for those of the fitted quadratic shapes, were found to be sufficiently small^18^, with peak-to-valley differences below 2 nm when the mirrors were not deformed. The central region (10 mm width) of each mirror was coated with a 100-nm-thick platinum layer on a thin chromium binding layer.

### Precise deformation procedure

To control the mirror shapes with 2 nm accuracy (*i.e*., *λ*/8), we used the following procedure with offline and online adjustments.

In the offline adjustment step, an optical Fizeau interferometer (VeriFire XPZ, Zygo Corp.) was used to monitor the mirror shape. Each mirror was deformed into the target ellipse with an accuracy of 2 nm by monitoring the mirror shape. During the offline adjustment step, the voltages applied to each of the electrodes were recorded in a log file.

**Figure 4:**
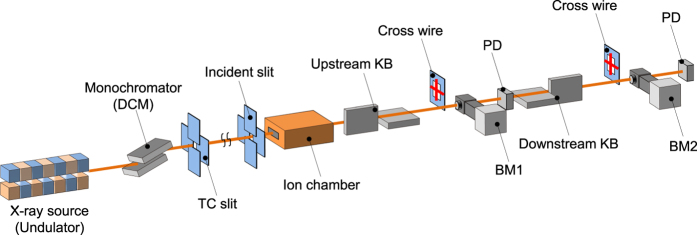
Experimental setup of focusing system. DCM: double-crystal monochromator. TC: transport channel. PD: photodiode. BM: beam monitor.

In the online adjustment step, the mirrors were deformed after being installed in the X-ray focusing system (Fig. 4), while monitoring their shapes with the pencil beam method^26^,^27^ (see Methods section). Before the fine adjustment process, voltage patterns identical to those used in the offline adjustment step were applied to the electrodes. The shapes could be reproduced with small deviations of less than ~50 nm. Then, the deformations of the upstream mirrors were finely adjusted while monitoring their shapes using the pencil beam method with the beam monitor located at the mid focus (BM1). After obtaining sufficiently small deformation errors, the downstream mirrors were finely adjusted in the same manner using the beam monitor placed at the final focus (BM2). Performing the pencil beam method with BM2 yielded information that included the shape errors of both the upstream and downstream mirrors. In Mode III, only the curvatures of the downstream mirrors were adjusted by applying voltages to the back electrodes after adjusting the upstream mirrors, because their illuminated areas along the optical axis were limited to 10 mm (20 mm) for the vertical (horizontal) focusing mirrors and there were too few electrodes within the illuminated areas to produce shapes with the required complexity. The wavefronts of the whole focusing system were thus adjusted by changing the shapes of the upstream mirrors while monitoring the wavefronts using BM2, based on the concept of wavefront compensation^17^.

### Hard X-ray focusing with variable-NA function

An experiment was performed at BL29XUL of SPring-8^28^ to test the proposed system. The optical elements were installed on a large granite bench in the third experimental hutch, which is ~98 m away from the undulator light source (Fig. 4). The four mirrors were precisely aligned with manipulators and autocollimators with sufficient accuracies. The incident X-ray beam with a photon energy of 10 keV was monochromatized with a Si 111 double-crystal monochromator. A cross-slit (TC slit) with a width of less than 10 μm was placed downstream from the monochromator (*i.e.*, ~50 m upstream from the mirrors) to create a virtual small X-ray source. The total demagnification ratios were evaluated to be 12–117 and 33–167 in the vertical and horizontal directions, respectively, in Modes I–III. To illuminate only the effective areas of the upstream mirrors, a 320-μm-wide cross-slit (incident slit) was placed in front of the upstream KB mirrors. This slit was also used in the pencil beam method. The beam passing through the focusing system was characterized at the mid and final foci using the knife-edge scanning method^29^,^30^ (see Methods section). When changing the NA, a voltage pattern determined in the offline adjustment process was applied, and BM1 and the knife-edge scanning system were translated along the beam axis to follow the shift of the mid focus position. The beam was then characterized in the same manner at the mid and final foci. Mode I was set up first and was then transformed into Mode II. Finally, Mode III was assembled.

**Figure 5:**
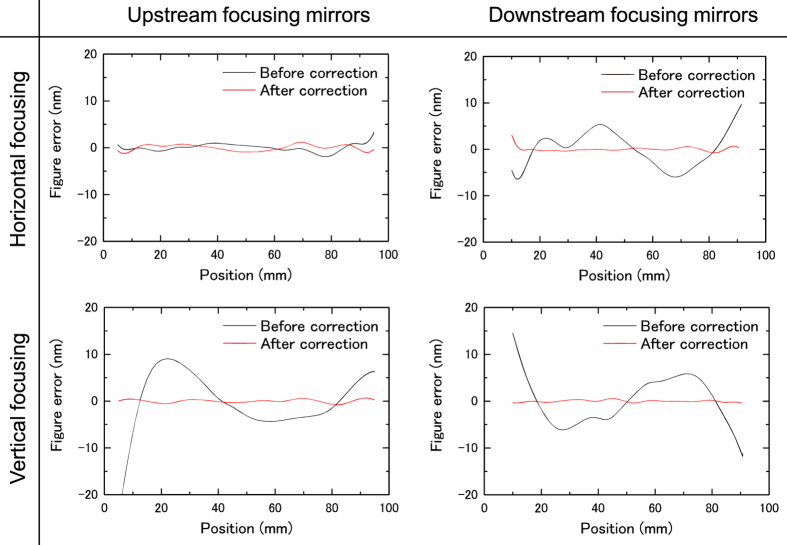
Typical deformation errors in Mode I before and after correction based on pencil beam method.

**Figure 6:**
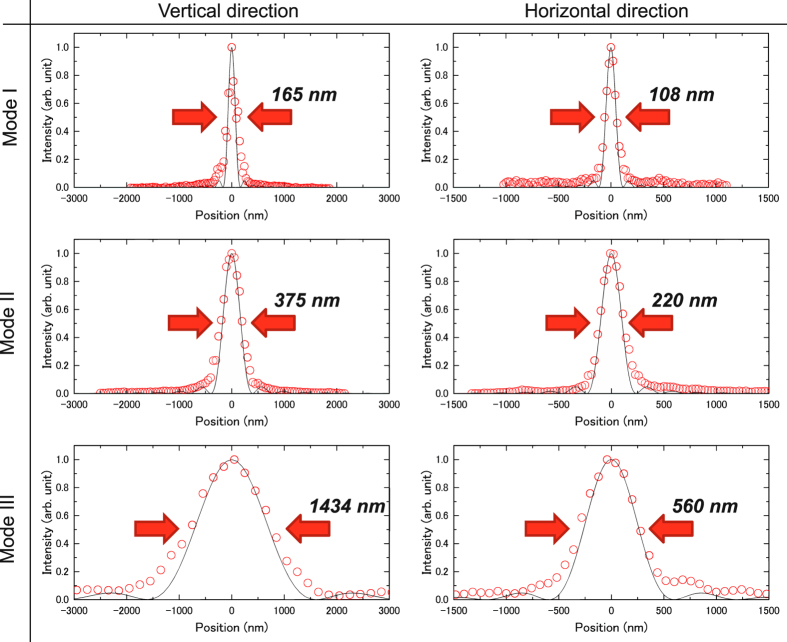
Intensity profiles of focused beams. Circles and solid lines show measured and calculated profiles, respectively. Small side lobes are observable, which might have resulted from mirror figure errors produced by slow PZT drift.

In Modes I–III, the mirrors were successfully deformed with an accuracy of ~2 nm. The typical shape errors after the correction using the pencil beam method in Mode I are shown in Fig. 5. Figure 6 presents the one-dimensional beam profiles obtained at the final foci in Modes I–III together with those calculated (solid lines) with our wave-optical simulator based on the Fresnel–Kirchhoff integral^31^. The obtained beam profiles have small side lobes almost symmetric to the main peak, in good agreement with the simulation. As can be seen from the widths of the main peaks, the beam size clearly changed as the NA was adjusted.

## Discussion

**Figure 7:**
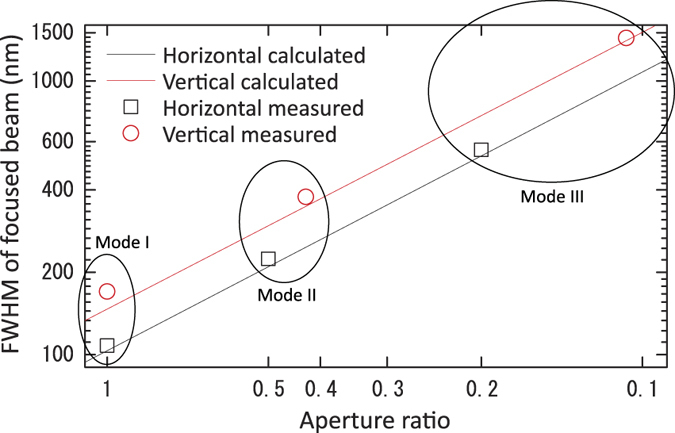
Relationship between FWHM and aperture ratio.

To illustrate the relationship between the NAs and the full widths at half-maximum (FWHMs) of the focused beam, Fig. 7 shows the calculated and experimentally measured FWHMs plotted against the aperture ratio. Both the experimental and calculated FWHMs are inversely proportional to the aperture ratio. The result shows that the beam size was successfully controlled by varying the NA of the adaptive focusing optics while keeping the diffraction-limited conditions and maintaining the positions of the foci. In addition, in Mode III, the errors in the downstream mirror shapes were successfully compensated for by adjusting the shapes of the upstream mirrors. This method will simplify the adjustment of two-stage adaptive focusing systems, leading to their practical application in the near future.

One of the remaining challenges in this focusing system is to increase the range in which the NA can be tuned. In this research, the ratio between the maximum and minimum NAs was approximately 10. This range can be expanded by thinning the substrates of the mirrors or by increasing the distance between the upstream and downstream mirrors. The former option is unsuitable because undesirable and unpredictable deformation will arise when mounting a thin mirror and contacting the electrodes to apply voltages. In addition, a focusing system with thin mirrors may be sensitive to vibration. However, the second option, increasing the distance between the mirrors, is acceptable. In this experiment, the distances between the upstream and downstream mirrors were 1.5 m (1.2 m) in the horizontal (vertical) direction due to limitations of the granite bench used for the optical system. If the distance were increased by a factor of 10, the range could be enlarged to ~100. Such a configuration can be applied for the two-stage focusing system^32^ in BL3 of SACLA, which has a large distance of 78 m between the upstream and downstream mirrors.

We expect that the proposed adaptive focusing system will be used in various multi-functional microscopes and advanced CDI in existing synchrotron radiation X-ray sources and advanced light sources, such as XFELs and ultimate storage rings.

## Methods

### Substrate surface shape correction

Substrate shape correction was performed by EEM with a computer control system^25^ after constructing the bimorph structure. A 10-mm-wide central region of each substrate was processed by EEM with a shape accuracy of approximately 2 nm, except for the second-order-polynomial shapes. The shapes were measured with at least 1 nm accuracy using our developed stitching interferometer^33^,^34^. The roughness of each processed surface was confirmed to be ~0.2 nm (root mean square) with a microscopic interferometer (NewView 5030, Zygo Corp.), which is sufficiently small to retain ideal reflectivity.

### Pencil beam method

The pencil beam method^26^,^27^ was used to determine the slope error distribution on a target focusing mirror using a slit and a beam monitor. Typically, ~20 μm X-ray beams passing through the slit placed immediately upstream of the target mirror illuminated one section of the mirror surface. The reflected beam position was detected using a beam monitor placed at the focus. This process was repeated until the entire effective area was scanned. Then, by calculating the median point shift of the reflected beam with sub-pixel accuracy, the slope error distribution over the scanned area was obtained. For this study, special beam monitors with high magnification, consisting of complementary metal–oxide–semiconductor (CMOS) cameras, thin scintillators, and lenses, were developed^18^. The detectable minimum median point shift was ~10 nm^18^. The pencil beam scan measurements of the deformable mirrors were repeated with a typical accuracy of ~50 nrad^18^.

### Focused beam characterization

Except for the profile at the mid focus in Mode III, the one-dimensional intensity profiles of the focused beams were measured by the dark-field knife-edge scanning method^29^. In this method, signals scattered from a knife edge are collected by blocking the bright field area with a slit. A 50-μm-diameter gold wire was employed as the knife edge in order to reduce the intensity of reflected X-rays from the wire surface. For the mid focus in Mode III, the intensity profile was obtained by the conventional knife-edge scanning method^30^ due to the space limitations. In this method, a 200-μm-diameter gold wire was used as the knife edge. The wires were scanned with a motorized feedback stage with 1 nm positioning accuracy, and the X-ray signals were detected with a PIN photodiode. Each beam was characterized several times using different scanning steps to reduce the measurement errors.

## Additional Information

**How to cite this article**: Matsuyama, S. *et al.* Nearly diffraction-limited X-ray focusing with variable-numerical-aperture focusing optical system based on four deformable mirrors. *Sci. Rep.* **6**, 24801; doi: 10.1038/srep24801 (2016).

## Figures and Tables

**Table 1:** Design parameters of the optics.

	Horizontal focusing	Vertical focusing
Source-1st mirror (mm)*	50000	50130
1st mirror-mid focus (mm)*		
Mode I	750	620
Mode II	1000	870
Mode III	1250	1120
1st mirror-2nd mirror (mm)*	1500	1240
2nd mirror-final focus (mm)*	300	430
Grazing-incidence angle (mrad)**		
Upstream mirror	4.0	4.0
Downstream mirror	4.0	4.0

^*^Distance between centres of mirrors.

^**^At centre of mirror.

**Table 2:** Optical parameters of Modes I, II, and III.

	Mode I		Mode II		Mode III	
Focusing direction*	H	V	H	V	H	V
NA (×10^−4^)	5.33	3.72	2.67	1.60	1.07	0.409
Aperture ratio	1.00	1.00	0.50	0.43	0.20	0.11
Ideal spot size (nm) in FWHM**	103	148	209	353	530	1423

^*^‘H’ and ‘V’ represent horizontal and vertical directions, respectively.

^**^The FWHMs were calculated with a wave-optical simulator based on the Fresnel–Kirchhoff integral.
